# Bladder paraganglioma managed with transurethral holmium laser resection: A case report

**DOI:** 10.1097/MD.0000000000026909

**Published:** 2021-08-27

**Authors:** Xin Zhu, Mi Zhou, Haitao Yu, Youlin Kuang, Yong Chen, Heqiu Li, Xin Gou

**Affiliations:** a Department of Urology; b Department of Respiratory and Critical Care Medicine, The First Affiliated Hospital of Chongqing Medical University; c Department of Pathology, Molecular Medicine Testing Center, Chongqing Medical University, Chongqing, China.

**Keywords:** bladder paraganglioma, case report, hypertension, transurethral holmium laser resection

## Abstract

**Rationale:**

Bladder paraganglioma is characterized by headache, palpitations, hypertension, blurred vision, or sweating during voiding. Transurethral holmium laser resection is a safe and efficacious alternative method for the resection of bladder neoplasms.

**Patient concerns:**

A 24-year-old female had a 2-year history of intermittent headaches, palpitation, and sweating during micturition.

**Diagnosis:**

Physical examination revealed a rise in the patient's blood pressure to 180/90 mmHg after micturition. Laboratory examination found that the blood catecholamine metabolites were significantly increased. Abdominal ultrasound and computed tomography (CT) scan indicated a 37 mm × 31 mm paraganglioma situated at the right anterolateral wall of the bladder. A diagnosis of bladder paraganglioma was considered based on a comprehensive evaluation of the physical examination, laboratory examination, ultrasound and computerized tomography scan.

**Interventions:**

Preoperative oral administration of a nonselective α-adrenergic receptor antagonist (phenoxybenzamine, 10 mg three times a day,) accompanied by a high-sodium diet and generous fluid intake, was initiated 2 weeks before the surgery to stabilize intraoperative hemodynamics. As the patient was newly married and nulligravid, management with transurethral resection was considered superior to open or partial cystectomy and was selected as the treatment method.

**Outcomes:**

Transurethral holmium resection of the bladder paraganglioma was successfully performed with blood loss less than 20 ml and well-controlled intraoperative blood pressure. The 1-year follow-up results demonstrated well-controlled symptoms. Cystoscopy and evaluation of blood catecholamine metabolites revealed no disease recurrence.

**Lessons:**

Transurethral holmium laser resection is a good alternative approach for the resection of bladder paraganglioma, given its advantages of safety and efficacy.

## 1 Introduction

Pheochromocytomas and paragangliomas are rare neuroendocrine tumors, characterized by the potential to secrete catecholamines from chromaffin tissues of the adrenal medulla and extra-adrenal sympathetic paraganglia, respectively.^[1,2]^ Although bladder paragangliomas account for less than 0.05% of all bladder tumors and less than 1% of all pheochromocytomas, the bladder remains the most commonly affected site in the genitourinary tract.^[3,4]^ Methods of surgical treatment of paraganglioma of the bladder include transurethral resection, partial cystectomy, and radical cystectomy. Although many studies have confirmed the effectiveness of transurethral resection, there is a lack of evidence regarding the rationale for choice of the appropriate surgical method for patients.^[5]^ Transurethral holmium laser resection had less intraoperative and postoperative complications, including obturator nerve reflex, transient hematuria and postoperative bladder irritation.^[6]^ In this report, we demonstrate the clinical features, pathological characteristics, and prognosis of a functional bladder paraganglioma, treated by transurethral holmium laser resection.

## 2 Case presentation

**Figure 1:**
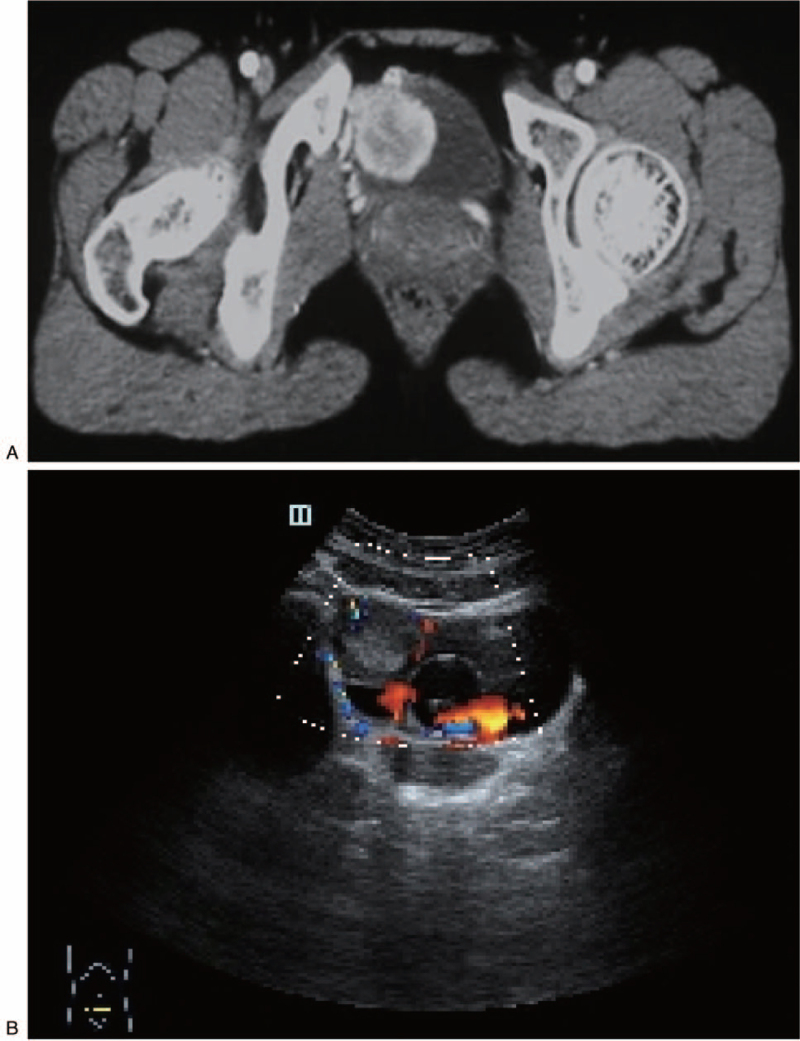
Images show a tumor at the right side of the anterior bladder wall. (A) Contrast-enhanced computed tomography revealed a mass in the bladder. (B) Ultrasound imaging revealed a mass in the bladder.

**Figure 2:**
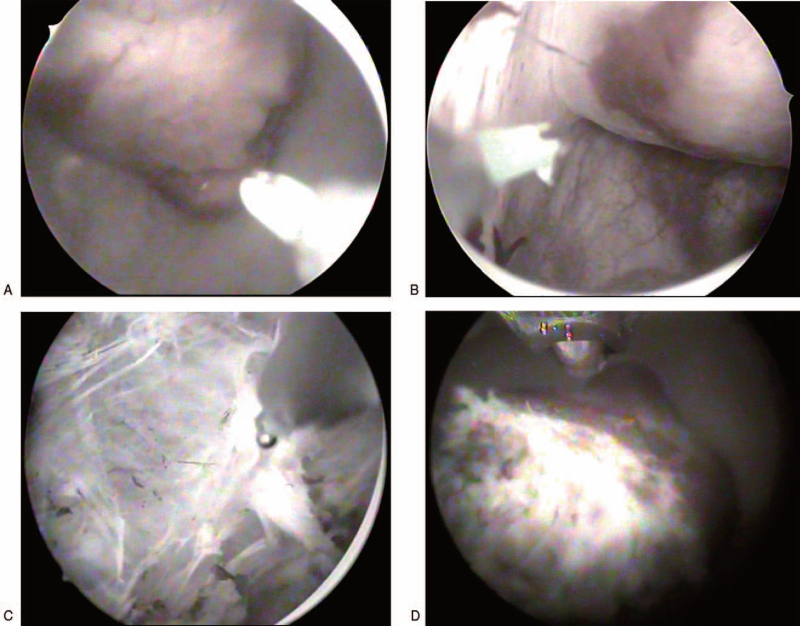
Intra-operative images of the bladder paraganglioma. (A) Endoscopic appearance of the tumor. (B) The base of the tumor. (C) The appearance of the bladder wall after transurethral holmium resection of the tumor. (D) The resected tumor for morcellation.

**Figure 3:**
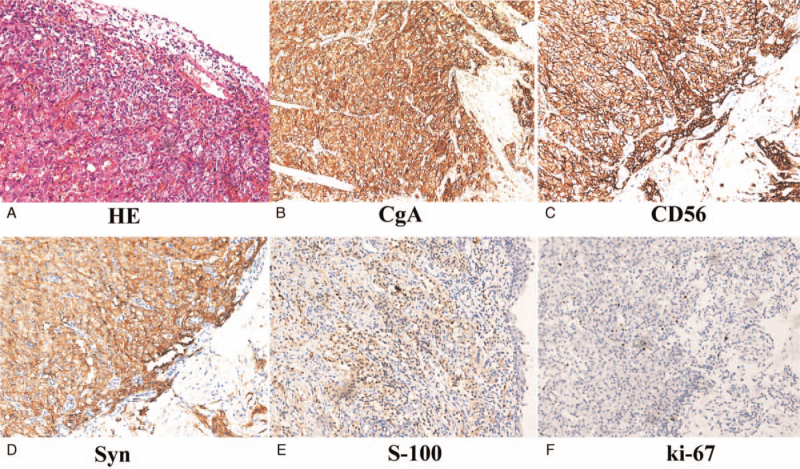
Pathology results of the bladder paraganglioma. (A) Hematoxylin-eosin staining × 200. (B) Immunostaining for chromogranin A was strongly positive. (C) Immunostaining for CD56 was strongly positive. (D) Immunostaining for synaptophysin was strongly positive. (E) Immunostaining for S-100 was positive. (F) Immunostaining for Ki67 was weakly positive.

**Figure 4:**
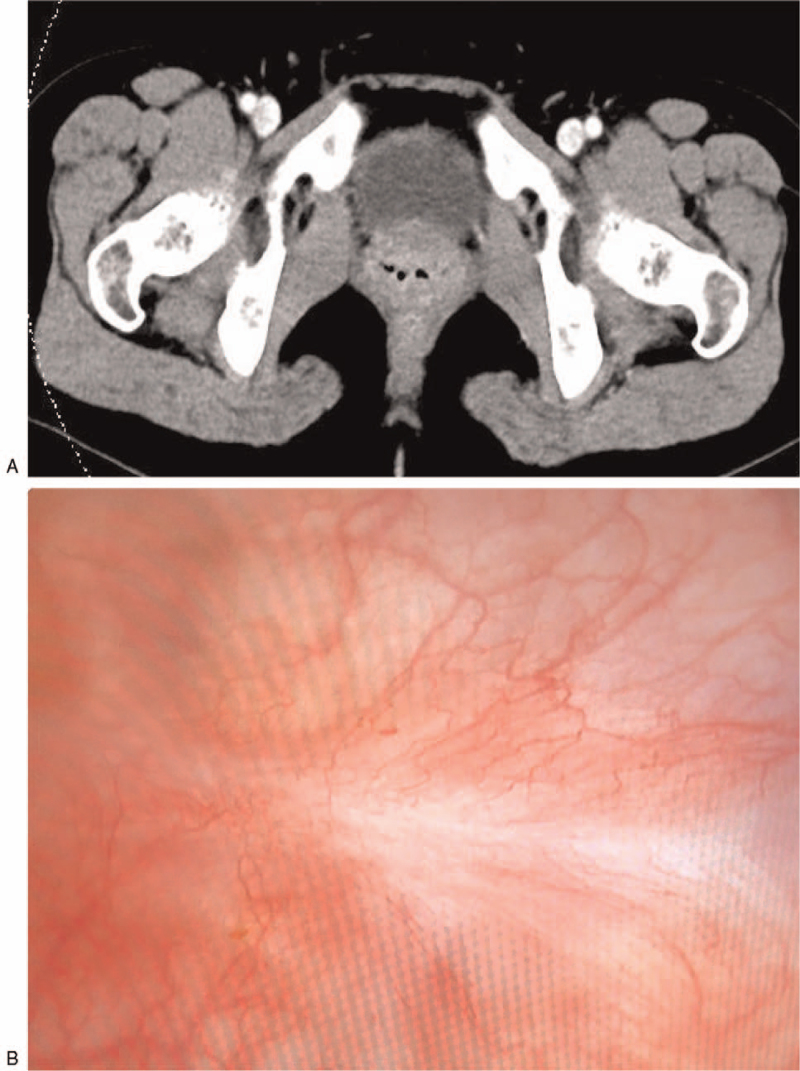
Postoperative pictures of the bladder paraganglioma. (A) Computed tomography of the bladder indicated no recurrence. (B) Endoscopic appearance of the bladder wall after transurethral holmium resection of the tumor.

A 24-year-old female was admitted to our hospital, complaining of a 2-year history of intermittent headaches, palpitation, and sweating during micturition without any past medical history or comorbidities. Two months before admission, physical examination revealed elevated blood pressure of 180/100 mmHg after micturition. Abdominal ultrasound and CT scan were performed, which demonstrated a 37 mm × 31 mm tumor located at the right anterolateral wall of the bladder, with intense early arterial phase enhancement (Fig. 1). A diagnosis of bladder paraganglioma was considered. After admission, blood catecholamine metabolites were detected to be increased (Table 1), and a final diagnosis of bladder paraganglioma was made. Surgical resection is the cornerstone of treatment for paragangliomas. As the patient was newly married and nulligravid, transurethral resection was a better choice than open or partial cystectomy. Preoperative oral administration of a nonselective α-adrenergic receptor antagonist (phenoxybenzamine, 10 mg three times a day), accompanied by a high-sodium diet and generous fluid intake, was initiated 2 weeks before the surgery. A transurethral holmium resection (Holmium 1.5 J∗25 Hz, and ND:YAG 100 W) of the bladder paraganglioma was successfully performed, with blood loss less than 20 ml. The operation took 46 min (Fig. 2), the blood pressure was well-controlled intra-operatively, and the resected tumor was morcellated by the transurethral method. Pathology results, including those of hematoxylin-eosin staining, positive immunostaining for chromogranin A, CD56, synaptophysin and S-100, as well as 3% positive percentage of Ki67, confirmed bladder paraganglioma (Fig. 3). One year after the operation, the patient remained asymptomatic with normal blood pressure and heart rhythm; all other examinations, including CT scan, cystoscopy, blood catecholamine metabolites and cystoscopy were normal (Fig. 4).

**Table 1:** The blood catecholamines metabolite levels of the patient.

The blood catecholamines metabolite levels	
Metanephrine	79.9 ng/L
Norepinephrine	637.5 ng/L
vanillylmandelic acid	45.7 ng/ml
Catecholamine	200.7 ng/ml

## 3 Discussion

Bladder paraganglioma is a rare subtype of bladder tumor, which was first reported by Zimmerman et al in 1953.^[7]^ It accounts for approximately 0.06% of bladder tumors and 1% of all pheochromocytomas.^[8]^ Literature reports the age of onset as 20–40 years, and a higher incidence in women than men.^[9]^ Case reports in Japan show that bladder paraganglioma commonly occurs at the following locations of the bladder: posterior wall (30.1%), dome (21.6%), anterior wall (19.1%), side wall (13.6%), triangle area (11.1%), and neck (3.7%). %).^[4,10]^

According to secretion of catecholamines by the tumor, bladder paragangliomas can be divided into functional and non-functional types.^[11,12]^ Functional bladder paraganglioma can manifest as clinical and resting types. Hypertension, dizziness, palpitations, and even syncope during urination are specific manifestations of the clinical type, owing to catecholamine secretion. The patient in this case demonstrated the clinical type of bladder paraganglioma. While the clinical symptoms of the resting type are concealed, it leads to the secretion of a large amount of catecholamines into the blood, which eventually cause symptoms. Patients exhibiting the resting type usually have microscopic or gross hematuria as the main clinical manifestations, and there are no symptoms such as hypertension caused by catecholamine secretion, which is detected only by imaging examinations.^[13]^

The preoperative qualitative diagnosis of bladder paraganglioma primarily relies on blood and urine catecholamine levels, 24 h urine vanillylmandelic acid, and other tests. Localization diagnosis relies on ultrasound, enhanced CT scan, and cystoscopy. Ultrasound is the most commonly used modality to diagnose bladder paraganglioma.^[4]^ Ultrasound examination revealed the tumor as a hypoechoic or isoechoic mass, and color Doppler ultrasound showed an abundant blood supply to the tumor. Bladder paraganglioma appears as a circular mass growing into the bladder on a plain CT image. The enhanced CT scan shows that the bladder paraganglioma is significantly enhanced in the arterial phase and weakened in the excretion phase. This is a typical feature, which is important for preoperative positioning and qualitative diagnosis.^[14]^ Cystoscopy can be used for complete visual inspection of the tumor. For patients with suspected bladder paraganglioma, high pressure on the bladder or squeezing the tumor during the cystoscopy should be avoided to prevent catecholamine secretion, which may lead to a hypertensive crisis. Bladder paraganglioma often appears as a single mass, which is significantly different from the cauliflower-like appearance commonly seen in transitional epithelial cancer. In early bladder paraganglioma, the surface of the tumor mucosa is usually normal on cystoscopy, and biopsy results are mostly negative. In advanced bladder paraganglioma, cystoscopy frequently reveals hyperemia, calcification, or necrosis of the bladder mucosa, which is difficult to distinguish from bladder cancer. According to previous studies, 61.6% of bladder paragangliomas confirmed by postoperative pathological diagnosis were misdiagnosed as bladder cancer or intramucosal bladder tumors, and only 28.9% of bladder paragangliomas were diagnosed preoperatively.^[15]^ In view of the high rate of misdiagnosis of bladder paraganglioma, its possibility should be considered before surgery in patients with atypical urothelial cancers, such as bladder submucosal tumors. For functional tumors, metaiodobenzylguanidine radionuclide imaging and positron emission tomography show better sensitivity and specificity, especially in the detection of multiple, metastatic, or recurrent lesions.^[3]^

The final diagnosis of bladder paraganglioma depends on histology and immunohistochemistry examination. Immunohistochemistry is positive for Cg A, Syn, S-100, CD56, and negative for CK and CEA in bladder paragangliomas.

The grading of adrenal pheochromocytoma and paraganglioma (GAPP) is a risk stratification tool used to predict metastasis and patient prognosis. The full score is 10 points, 0–2 scores are divided into well-differentiated type, 3–6 into moderately differentiated type, and 7–10 into poorly differentiated type. Approximately 68% of paragangliomas are well-differentiated, with metastatic and 5-year survival rates of 3.6% and 100%, respectively; approximately 22% are moderately differentiated, with a metastatic and 5-year survival rates of 60% and 66.8%, respectively; and approximately 10% are poorly differentiated, with metastatic and 5-year survival rates of 88.2% and 22.4%, respectively.^[16]^

Surgical resection is the first choice of treatment for bladder paraganglioma. Commonly used surgical methods include partial cystectomy, transurethral resection and radical cystectomy. The choice of surgical method should be based on the specifics of the patient and the technical strength of the operator, in order to reduce the risk and maximize patient protection. Partial cystectomy has the advantages of less interference to tumors, relatively stable intraoperative blood pressure, and low recurrence rate. With the advancement of laparoscopic technology over recent years, laparoscopic and robot-assisted laparoscopic partial cystectomy have the potential to replace traditional open surgery, being a safe and promotion-worthy choice.^[17]^ Transurethral resection has the advantages of less trauma and reproducibility; however, its application in the treatment of clinical bladder paraganglioma is still controversial. According to the study by Pahwa et al, high pressure on the bladder and tumor resection during the operation would lead to the release of a large amount of catecholamines, resulting in dramatic fluctuations in blood pressure. Moreover, these tumors are mostly located in the muscular layer, and the intraoperative resection might not be complete, increasing the probability of tumor recurrence.^[18]^

Considering the newly-married, nulligravid female, transurethral resection was a better option than partial or complete cystectomy. Compared to traditional transurethral resection with electrocision, transurethral resection with holmium-yag is endowed with lack of obturator reflex, good bleeding control, and clear demarcation of anatomical relationships between the tumor and muscular fibers. Although the 1-year follow-up including blood catecholamine metabolite levels, pelvic CT scan, and cystoscopy revealed satisfactory results, a life-long follow-up is still required to monitor recurrence. Some previously reported cases of bladder paraganglioma are summarized in Table 2. To our best knowledge, this report was the first one to illustrate the application of transurethral holmium laser resection in bladder paraganglioma. We recommend transurethral holmium laser resection for bladder paragangliomas that are anatomically confined to the submucosa, protruding into the bladder, have a small tumor volume (<4 cm), and good accessibility (Non-ureteral orifice area, anterior bladder, top wall, etc.).

**Table 2:** Summary of previous case reports.

Year	First author	Tittle	Case presentation	Intervention	Follow-up and Outcome
2013	Michelle Christodoulidou	Incidental paraganglioma of the urinary bladder in a 66-year-old woman	Age: 66-year-old. Gender: female. Bladder mass: base of the bladder (7 mm in diameter)	TURBT	One year follow-up without recurrence
2017	Genta Iwamoto	Paraganglioma in the bladder: a case report	Age: 77-year-old. Gender: male. Bladder mass: front wall of bladder (26 mm in diameter)	TURBT	Died 8 months after TURBT due to aspiration pneumonitis
2015	Arindam Bagchi	Urinary Bladder Paraganglioma presenting as Micturition-Induced Palpitations, Dyspnea, and Angina	Age: 45-year-old. Gender: female. Bladder mass: anterior bladder wall (2.4 × 3.5 cm)	TURBT	Long-term follow-up without recurrence
2019	Baomin Qiao	Non-functional paraganglioma of urinary bladder managed by transurethral resection	Age: 44.5 ± 13.6 years (range 29–70 years). Gender: 4 male, 6 female. Bladder mass: 1.5 cm x 1.3 cm to 3.5 cm x 2.1cm	TURBT	Follow-up period: 36.4 ± 24.8 months (range, 8–95 months). One case of T2 relapsed on the 37th month
2020	Gil Falcão	Bladder paraganglioma: a case report	Age:53-year-old. Gender: male. Bladder mass: lateral right wall	TURBT Radical cistoprostatectomy (After one month)	After 4 years of follow-up without recurrence

TURBT = transurethral resection of bladder tumour.

## Acknowledgments

We acknowledge patient for her cooperation and trust to our therapy.

## Author contributions

**Conceptualization:** Xin Gou.

**Data curation:** Xin Zhu, Mi Zhou, Haitao Yu, Youlin Kuang, Yong Chen, Heqiu Li.

**Methodology:** Xin Zhu, Youlin Kuang, Yong Chen, Heqiu Li.

**Writing – original draft:** Xin Zhu, Mi Zhou, Haitao Yu.

**Writing – review & editing:** Xin Gou.
